# Clinical Efficacy of Qili Qiangxin Capsule Combined with Western Medicine in the Treatment of Chronic Heart Failure: A Systematic Review and Meta-Analysis

**DOI:** 10.1155/2021/9761159

**Published:** 2021-08-09

**Authors:** Xiaoming Xu, Yang Yang, Gendong Zhou, Zhengxuan Du, Xiaohong Zhang, Wei Mao, Hongwen Cai

**Affiliations:** ^1^Department of Cardiology, The First Affiliated Hospital of Zhejiang Chinese Medical University, Hangzhou, Zhejiang, China; ^2^Department of Cardiology, The Third Affiliated Hospital of Anhui Medical University, Hefei 230061, Anhui, China

## Abstract

Qili Qiangxin capsule (QQC) is a formulation of traditional Chinese medicine commonly used for the treatment of heart failure in China. This meta-analysis aimed to assess the clinical efficacy of QQC combined with western medicine in the treatment of chronic heart failure (CHF). We conducted a systematic review and meta-analysis abided by the PRISMA guidelines. Literature search was conducted in the China National Knowledge Infrastructure, Wanfang Database, Chinese Scientific Journals Database, PubMed, and Web of Science from inception to August 2020. A total of 52 eligible studies were obtained, and 42 of these studies were included in the meta-analysis. The results showed that, compared with western medicine alone, the combination of Qili Qingxin capsule and Western medicine treatment has better efficacy (metoprolol: RR: 1.24, 95%CI 1.14–1.34; carvedilol: RR: 1.24, 95%CI 1.14–1.34; trimetazidine: RR: 1.20, 95%CI: 1.12–1.27; sacubitril valsartan sodium: RR: 1.23, 95%CI: 1.11–1.36; sodium nitroprusside: RR: 1.33, 95%CI: 1.23–1.45; and bisoprolol: RR: 1.31, 95%CI: 1.15–1.49) and increased the level of LVEF, LVEDD, and 6MWT of patients with CHF and reduced the adverse effects and the level of HR, LVESD, BNP, and Hs-cTnT as well. However, there is high heterogeneity in the meta-analysis of LVEDV, BNP, NT-proBNP, Hs-cTnT, 6MWT, and adverse effects, and the methodological quality of the included studies was poor. Therefore, further studies with good methodological quality and large sample size are required to validate our findings. In our study, evidence suggests that Qili Qiangxin capsule combined with Western medicine may improve therapeutic effect and the quality of life of patients with CHF.

## 1. Introduction

Chronic heart failure (CHF) has been recognized as one of the major global health issue which is associated with high risk of morbidity, mortality, and cost [1]. According to the survey, there are 23 million patients with CHF worldwide, approximately 5.7 million in the US and over 4.2 million in China [1]. The pathogenesis of CHF mainly includes primary myocardial damage resulted from long-term glucose and lipid metabolism disorder and ischemic myocardial damage [2]. Especially, the elderly showed higher morbidity of CHF [3]. In the future, the number of patients with CHF would increase significantly with the aging of the population [4].

Conventional Western medicine for CHF treatment includes beta-blockers, cardiotonic steroids, diuretics, and angiotensin-converting enzyme inhibitors (ACEI). Although these treatments have certain therapeutic effects, such as a reduction of heart rate and left ventricular filling pressure and an improvement in exercise capacity [4], an improvement in the curative effect is still necessary because of the adverse effects and short-term effects. For example, the metoprolol, a beta-blocker, may cause depression and anxiety during the CHF treatment [5]. With the development of science and technology, more and more anti-CHF ingredients have also been discovered [6–8]. Complementary and alternative medicine therapies have become increasingly popular and are used regularly by patients with chronic disorders [9]. Currently, the combination of traditional Chinese medicine (TCM) and western medicine has been widely used for the CHF treatment [10–12]. According to the perspective of TCM, the etiology of CHF is the yang deficiency of heart which is the result of the inadequacy of Qi and blood, blood stasis, and phlegm-dampness. With the in-depth research of TCM, the main CHF treatment is replenishing Qi and nourishing Yin [13].

Qili Qiangxin capsule (QQC) is a traditional Chinese herbal remedy that was mentioned as a treatment with certain therapeutic effects, such as reducing heart rate and revering ventricular remodeling, for the CHF by China Food and Drug Administration [14]. QQC is a standardized Chinese herbal extract prepared from 11 Chinese herbs, including dry root of *Astragalus* membranaceus (Fisch.) Bge. var. mongholicus (Bge.), Hsiao or *Astragalus* membranaceus (Fisch.) Bge., dry root of *Panax* ginseng C. A. Meyer, *Aconitum* carmichaeli Debx., dry root of *Salvia* miltiorrhiza Bunge, dry seeds of *Lepidium* apetalum Willd. or *Descurainia* Sophia (L.) Webb ex prantl., dry tubers of *Alisma* orientalis (Sam.) Juzep., dry rhizome of Polygonatum odoratum (Mill.) Druce, dry twigs of *Cinnamomum* cassia Presl, dry flower of *Carthamus* tinctorius L., dry root bark of Periploca sepium Bge., and dried ripe peel of *Citrus* reticulata Blanco. Previous studies have reported that QQC played a role in the treatment of CHF through multiple mechanisms, such as reducing cardiac fibrosis remolding, improving cardiac function, reducing NT-proBNP, and regulating the inflammatory responses [13–16]. In addition, the efficacy and safety also have been proved in a study of the effects of QQC on 512 patients with CHF [17].

Currently, QQC combined with Western medicine has been widely used for the treatment of CHF. A lot of studies have reported that QQC combined with Western medicine can improve clinical outcomes compared to Western medicine treatment [18–20]. Sun et al. performed a meta-analysis in 2016 and concluded that QQC plus conventional treatment showed better efficiency than conventional treatment alone in the treatment of heart failure [21]. However, a subgroup analysis based on conventional treatments was not conducted in the meta-analysis. With the deepening of research on QQC in CHF in recent years, it is necessary to conduct an in-depth analysis of the clinical effects of QQC plus Western medicine on CHF patients. Given the small sample sizes and inconsistent results of previous research studies, this study aimed at conducting a systematic review and meta-analysis of the efficacy and safety of QQC combined with Western medicine in the treatment of CHF and providing reference for clinical diagnosis and treatment.

## 2. Method

This study was compiled based on the Preferred Reporting Items for Systematic reviews and Meta-Analyses statement (PRISMA) (Supplementary Materials S1) [22].

### 2.1. Search Strategy

We searched the following five databases from their start date to August 2020: China National Knowledge Infrastructure (CNKI), Wanfang Database, Chinese Scientific Journals Database (VIP), PubMed, and Web of Science. We used the following keywords and medical subject heading terms: (“Qili Qiangxin” OR “qiliqiangxin” OR “qiangxinli”) and (“Chronic heart failure” OR “Chronic cardiac failure” OR “Chronic heart decompensation”). We also hand-searched the reference lists of all full text papers for additional relevant reports. We did not impose any language restrictions.

### 2.2. Eligibility and Exclusion Criteria

Trials were considered eligible if they were (a) randomized controlled trials (RCTs); (b) enrolled participants with CHF; (c) compared the curative effects of the combination of QQC and Western drugs with Western drugs alone. The following studies were excluded, including repeated publication, literature with incomplete or incorrect data, and the control group without a description of the Western drugs used in this study.

### 2.3. Study Selection

At first, the duplicates of all records obtained from the electronic databases were removed. Then, two researchers read the titles and abstracts independently to identify the eligible studies. Full texts of the studies were read to determine whether they met the eligibility criteria. The disagreements were resolved by discussing with a third researcher.

### 2.4. Outcomes

Our primary outcome measures were the clinical efficiency. Secondary outcomes measures were heart rate (HR), left ventricular ejection fractions (LVEF), left ventricular end-diastolic dimension (LVEDD), left ventricular end-diastolic volume (LVEDV), left ventricular end-systolic diameter (LVESD), brain natriuretic peptide (BNP), N-terminal Pro B-type natriuretic peptide (NT-proBNP), high-sensitivity cardiac troponin T (Hs-cTnT), 6-min walk test (6MWT). We also collected adverse events data. RCTs reporting one or more of these outcomes were included.

### 2.5. Data Extraction and Quality Assessment

The following details were extracted by two researchers independently from included studies: first author; year; sample size; age; gender; course of treatment; the intervention of experimental group; the intervention of control group; and outcome data. The methodological quality of the included studies was used Cochrane 5.1.0 assessment tool. It includes following seven aspects: (1) random sequence generation; (2) allocation concealment; (3) blinding of participants and personnel; (4) blinding of outcome assessment; (5) incomplete outcome data; (6) selective reporting; and (7) other bias. Each domain of the included studies was evaluated as having a low, high, or unclear risk of bias. It was also independently completed by two researchers. The disagreements were resolved by discussing with a third researcher.

### 2.6. Data Analysis

The meta-analysis was performed by using RevMan 5.3 software. The relative risk (RR) and 95% confidence intervals (95% CI) were used for dichotomous variables, and the mean difference (MD) and 95% CI were used for continuous variables. This study used *I*^2^ and *Q* tests to evaluate heterogeneity of study. When the *I*^2^ >50% or *P* < 0.01 in the *Q* test, the random model would be used for meta-analysis. Otherwise, we used fixed model for meta-analysis.

## 3. Results

### 3.1. Description of Studies

**Figure 1:**
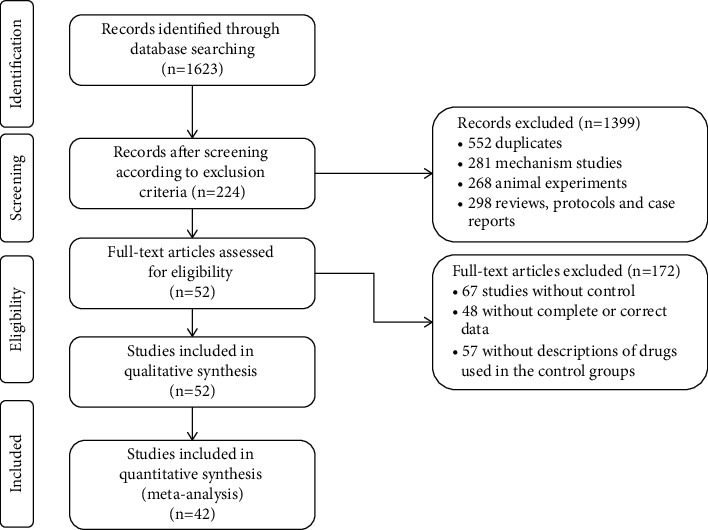
Flow chart of study searching and selection.

As shown in Figure 1, a total of 1623 studies were obtained from five databases. After removing the 552 duplicates, 281 mechanism studies, 268 animal experiments, 298 reviews, protocols, and case reports, we included 224 studies. By screening the full text articles, we removed 67 studies of no control group, 48 studies of incomplete or incorrect data, 57 studies of the control group without a description of the Western drugs used in study. Finally, we included 52 studies for the quality assessment, and 42 of these studies were included for meta-analysis.

### 3.2. Characteristics of Study

The characteristics of the 52 included studies are shown in Table 1. All included studies were conducted in China and published in Chinese. Among the included studies, 11 studies [18–20, 37, 40, 45, 46, 57–60] were related to clinical efficacy of QQC combined with metoprolol. 3 studies [36, 42, 53] were related to clinical efficacy of QQC combined with bisoprolol. 3 studies [51, 69, 70] were related to clinical efficacy of QQC combined with levocarnitine oral solution. 5 studies [31, 43, 44, 55, 56] were related to clinical efficacy of QQC combined with carvedilol. 10 studies [24, 26, 27, 32–35, 47–49] were related to clinical efficacy of QQC combined with trimetazidine. 1 study [23] was related to clinical efficacy of QQC combined with digoxin. 2 studies [54, 64] were related to clinical efficacy of QQC combined with coenzyme Q10. 1 study [65] was related to clinical efficacy of QQC combined with enalapril maleate and folic acid tablets. 4 studies [61, 66–68] were related to clinical efficacy of QQC combined with sacubitril valsartan sodium tablets. 1 study [30] was related to clinical efficacy of QQC combined with benazepril hydrochloride. 9 studies [25, 28, 29, 38, 39, 41, 52, 63, 71] were related to clinical efficacy of QQC combined with sodium nitroprusside, and 2 studies [50, 62] were elated to clinical efficacy of QQC combined with ivabradine.

### 3.3. Risk of Bias

**Figure 2:**
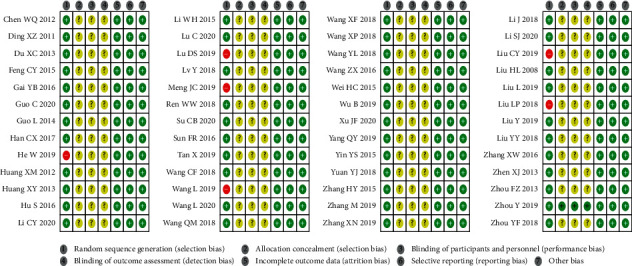
Risk of bias summary.

As shown in Figure 2, for the random sequence generation, 6 studies were high risk of bias, and the rest were low risk of bias. One study specified the methods of allocation, and the rest were unclear. One study blinded participants, personnel, and outcome assessment, whereas the rest had no description about the methods of the blinding. There were complete data in all studies, and all studies had low risk of bias in selective reporting and other source of bias.

### 3.4. Clinical Efficacy

**Figure 3:**
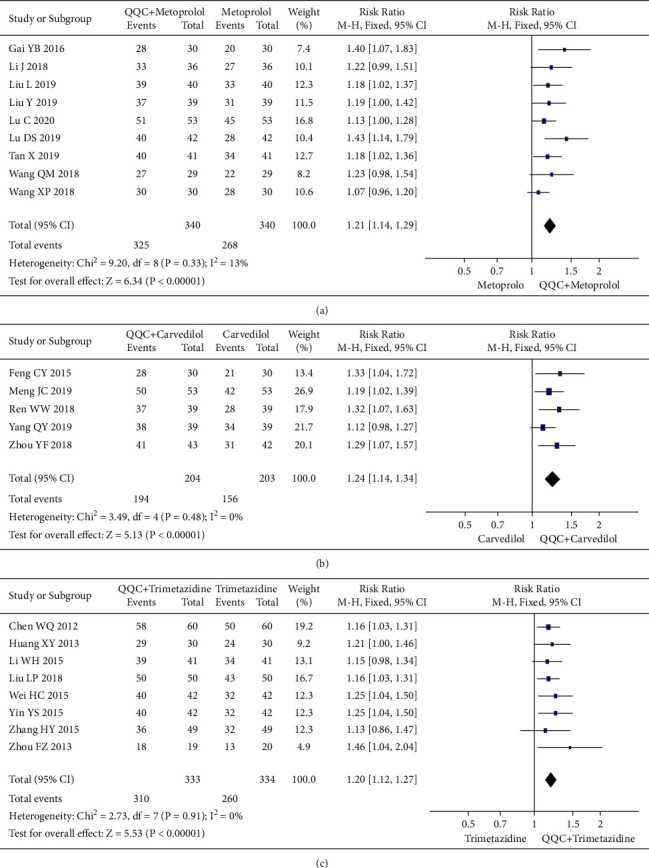
Forest plot of QQC plus Western medicine treatment versus Western medicine only for clinical efficiency (part 1). QQC: Qili Qiangxin capsule.

**Figure 4:**
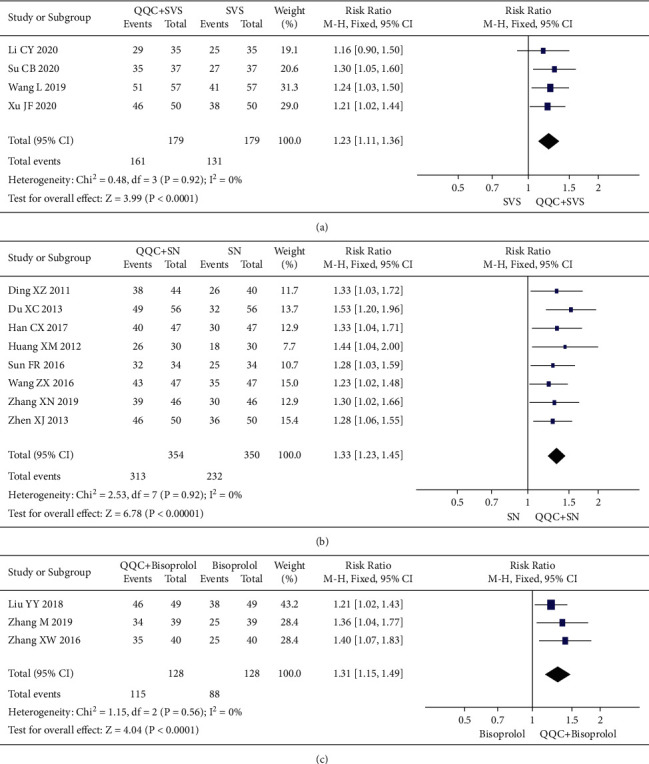
Forest plot of QQC plus Western medicine treatment versus Western medicine only for clinical efficiency (part 2). QQC: Qili Qiangxin capsule, SVS: aacubitril valsartan sodium, and SN: sodium nitroprusside.

**Figure 5:**
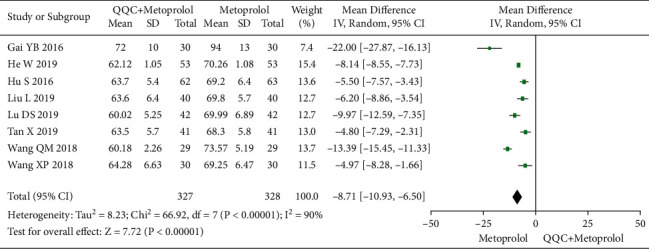
Forest plot of QQC plus Western medicine treatment versus Western medicine only for HR. QQC: Qili Qiangxin capsule, HR: heart rate.

In Figures 3 and 4, the meta-analysis results showed that the clinical efficacy of QQC plus Western drugs treatment was significantly better than Western drugs treatment alone. The following data showed the meta-analysis of QQC combined with different Western drugs treatment: (1) metoprolol (*n* = 9 trials, RR: 1.21, 95%CI: 1.14 to 1.29, Figure 3(a)); (2) carvedilol (*n* = 5 trials, RR: 1.24, 95%CI 1.14 to 1.34, Figure 3(b)); (3) trimetazidine (*n* = 8 trials, RR: 1.20, 95%CI: 1.12 to 1.27, Figure 3(c)); (4) sacubitril valsartan sodium (*n* = 4 trials, RR: 1.23, 95%CI: 1.11 to 1.36, Figure 4(a)); (5) sodium nitroprusside (*n* = 8 trials, RR: 1.33, 95%CI: 1.23 to 1.45, Figure 4(b)); and (6) bisoprolol (*n* = 3 trials, RR: 1.31, 95%CI: 1.15 to 1.49, Figure 4(c)).

### 3.5. Heart Rate (HR)

The meta-analysis with a random model showed that the HR of QQC plus metoprolol treatment was significantly lower than metoprolol treatment alone (*n* = 8 trials, MD: −8.71, 95%CI: −10.93 to −6.50, *P* < 0.00001, heterogeneity *χ*^2^ = 66.92, *P* < 0.00001, I^2^ = 90, Figure 5).

### 3.6. Left Ventricular Ejection Fractions (LVEF)

**Figure 6:**
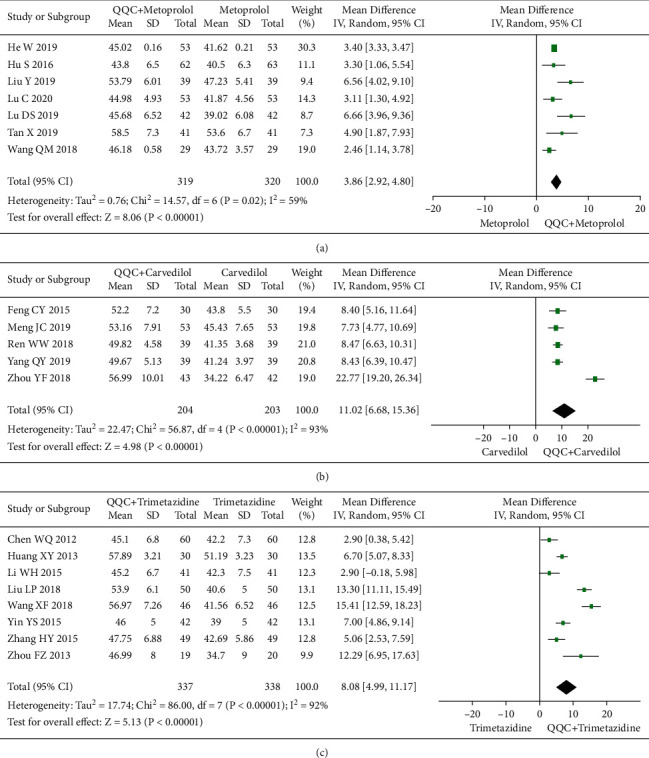
Forest plot of QQC plus Western medicine treatment versus Western medicine only for LVEF (part 1). QQC: Qili Qiangxin capsule, LVEF: left ventricular ejection fractions.

**Figure 7:**
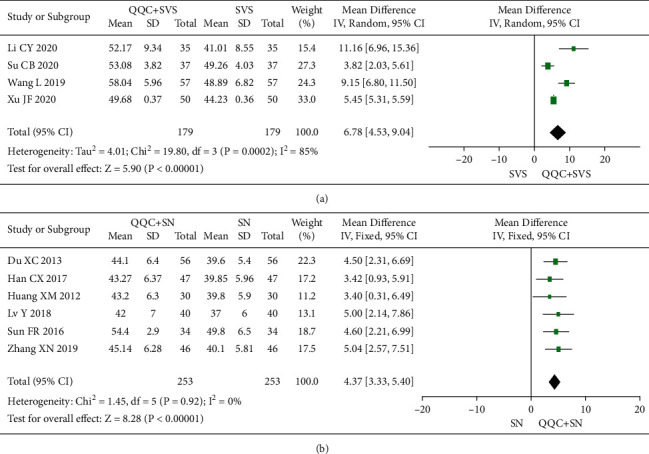
Forest plot of QQC plus Western medicine treatment versus Western medicine only for LVEF (part 2). QQC: Qili Qiangxin capsule, SVS: sacubitril valsartan sodium, SN: sodium nitroprusside, LVEF: left ventricular ejection fractions.

The meta-analysis results showed that the LVEF of QQC plus Western drugs treatment was significantly higher than Western drugs treatment alone (Figures 6 and 7). The following data showed the meta-analysis of QQC combined with different Western drugs treatment: (1) metoprolol (*n* = 7 trials, MD: 3.86, 95%CI: 2.92 to 4.80, Figure 6(a)); (2) carvedilol (*n* = 5 trials, MD: 11.02, 95%CI: 6.68 to 15.36, Figure 6(b)); (3) trimetazidine (*n* = 8 trials, MD: 8.08, 95%CI: 4.99 to 11.17, Figure 6(c)); (4) sacubitril valsartan sodium (*n* = 4 trials, MD: 6.78, 95%CI: 4.53 to 9.04, Figure 7(a)); (5) sodium nitroprusside (*n* = 6 trials, MD: 4.37, 95%CI: 3.33 to 5.40, Figure 7(b)).

### 3.7. Left Ventricular End-Diastolic Dimension (LVEDD)

**Figure 8:**
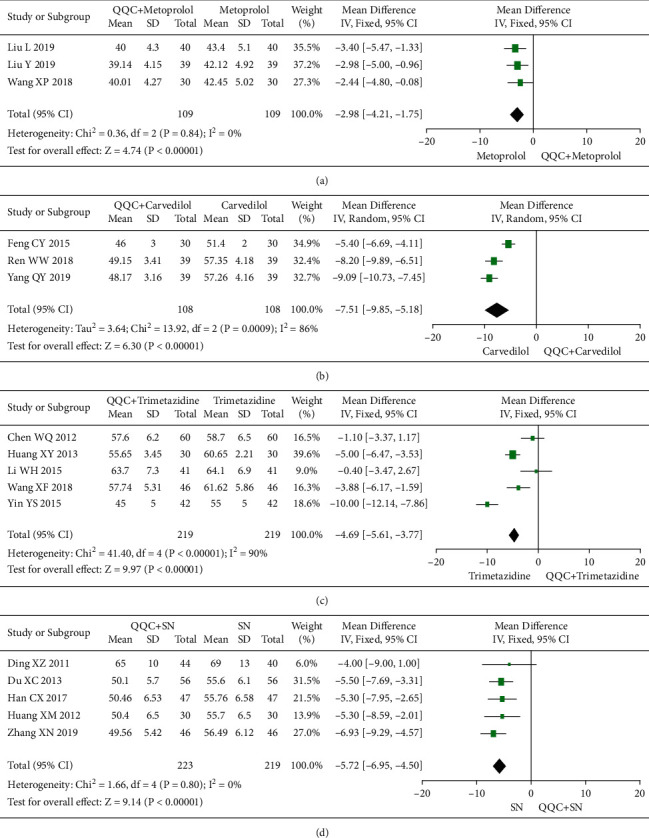
Forest plot of QQC plus Western medicine treatment versus Western medicine only for LVEDD. QQC: Qili Qiangxin capsule, SN: sodium nitroprusside, LVEDD: left ventricular end-diastolic dimension.

The meta-analysis results showed that the LVEDD of QQC plus Western drugs treatment was significantly higher than Western drugs treatment alone (Figure 8). The following data showed the meta-analysis of QQC combined with different Western drugs treatment: (1) metoprolol (*n* = 3 trials, MD: −2.98, 95%CI: −4.21 to −1.75, Figure 8(a)); (2) carvedilol (*n* = 3 trials, MD: −7.51, 95%CI: −9.85 to −5.81, Figure 8(b)); (3) trimetazidine (*n* = 5 trials, MD: −4.61, 95%CI: −7.26 to −1.07, Figure 8(c)); (4) sodium nitroprusside (*n* = 5 trials, MD: −5.72, 95%CI: −6.95 to −4.50, Figure 8(d)).

### 3.8. Left Ventricular End-Diastolic Volume (LVEDV)

**Figure 9:**
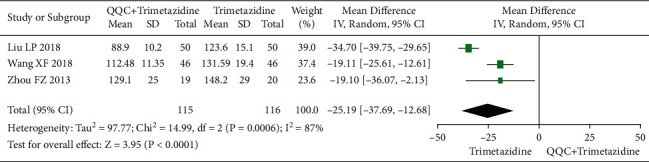
Forest plot of QQC plus Western medicine treatment versus Western medicine only for LVEDV. QQC: Qili Qiangxin capsule, LVEDV: left ventricular end-diastolic volume.

The meta-analysis with a random model showed that the LVEDV of QQC plus trimetazidine treatment was significantly lower than trimetazidine treatment alone (*n* = 3 trials, MD: −25.19, 95%CI: −37.69 to −12.68, *P* < 0.0001, heterogeneity *χ*^2^ = 14.99, *P*=0.0006, *I*^2^ = 87%, Figure 9).

### 3.9. Left Ventricular End-Systolic Diameter (LVESD)

**Figure 10:**
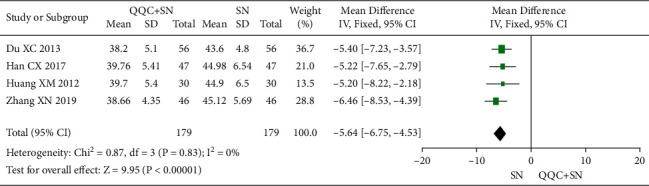
Forest plot of QQC plus Western medicine treatment versus Western medicine only for LVESD. QQC: Qili Qiangxin capsule, SN: sodium nitroprusside, LVESD: left ventricular end-systolic diameter.

The meta-analysis with a random model showed that the LVESD of QQC plus sodium nitroprusside treatment was significantly lower than sodium nitroprusside treatment alone (*n* = 4 trials, MD: −5.64, 95%CI: −6.75 to −4.53, *P* < 0.0001, heterogeneity *χ*^2^ = 0.87, *P*=0.83, *I*^2^ = 0%, Figure 10).

### 3.10. Brain Natriuretic Peptide (BNP)

**Figure 11:**
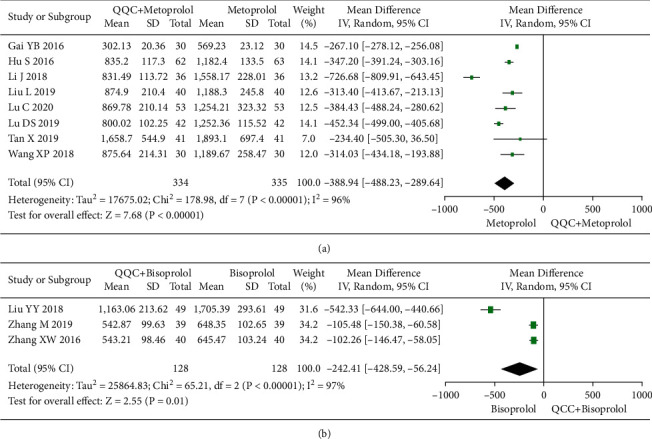
Forest plot of QQC plus Western medicine treatment versus Western medicine only for BNP. QQC: Qili Qiangxin capsule, BNP: brain natriuretic peptide.

The meta-analysis results showed that the BNP of QQC plus Western drugs treatment was significantly lower than Western drugs treatment alone (Figure 11). The following data showed the meta-analysis of QQC combined with different Western drugs treatment: (1) metoprolol (*n* = 8 trials, MD: −388.94, 95%CI: −488.23 to −289.64, Figure 11(a)) and (2) bisoprolol (*n* = 3 trials, MD: −242.41, 95%CI: −428.59 to −56.24, Figure 11(b)).

### 3.11. N-Terminal Pro-B-Type Natriuretic Peptide (NT-ProBNP)

**Figure 12:**
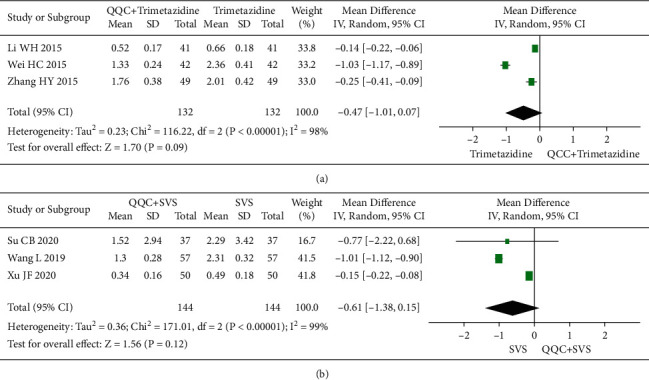
Forest plot of QQC plus Western medicine treatment versus Western medicine only for NT-proBNP. QQC: Qili Qiangxin capsule, SVS: sacubitril valsartan sodium, NT-proBNP: N-terminal pro-B-type natriuretic peptide.

The meta-analysis results showed that there was no significant difference between the NT-proBNP of QQC plus Western drugs treatment and Western drugs treatment alone (Figure 12). The following data showed the meta-analysis of QQC combined with different Western drugs treatment: (1) trimetazidine (*n* = 3 trials, MD: −0.47, 95%CI: −1.01 to 0.07, Figure 12(a)) and (2) sacubitril valsartan sodium (*n* = 3 trials, MD: −0.61, 95%CI: −1.38 to 0.15, *P*=0.12, heterogeneity *χ*^2^ = 171.01, Figure 12(b)).

### 3.12. High-Sensitivity Cardiac Troponin T (Hs-cTnT)

**Figure 13:**
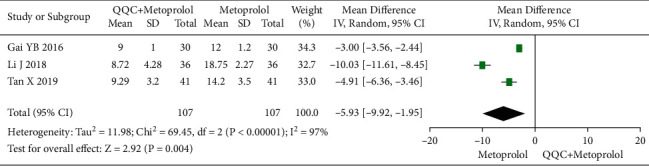
Forest plot of QQC plus Western medicine treatment versus Western medicine only for Hs-cTnT. QQC: Qili Qiangxin capsule, Hs-cTnT: high-sensitivity cardiac troponin T.

The meta-analysis with a random model showed that the Hs-cTnT of QQC plus metoprolol treatment was significantly lower than metoprolol treatment alone (*n* = 3 trials, MD: −5.93, 95%CI: −9.92 to −1.95, *P*=0.004, heterogeneity *χ*^2^ = 69.45, *P* < 0.0001, *I*^2^ = 97%, Figure 13).

### 3.13. 6-Min Walk Test (6MWT)

**Figure 14:**
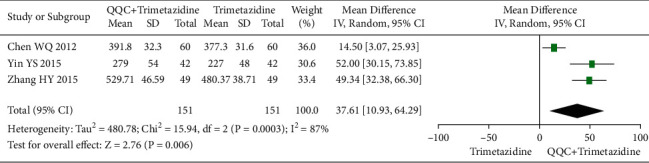
Forest plot of QQC plus Western medicine treatment versus Western medicine only for 6MWT. QQC: Qili Qiangxin capsule, 6MWT: 6-min walk test.

The meta-analysis with a random model showed that the 6MWT of QQC plus trimetazidine treatment was higher than trimetazidine treatment alone (*n* = 3 trials, MD: 37.61, 95%CI: 10.93 to 64.29, *P*=0.006, heterogeneity *χ*^2^ = 15.94, *P*=0.0003, *I*^2^ = 87%, Figure 14).

### 3.14. Adverse Effects

**Figure 15:**
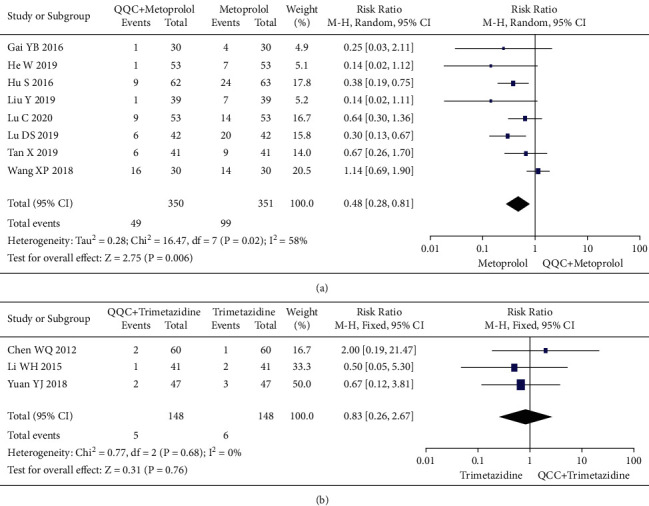
Forest plot of QQC plus Western medicine treatment versus Western medicine only for adverse effects. QQC: Qili Qiangxin capsule.

The adverse effects occurred in the included studies included headache, hypotension, hyponatremia, nausea, cough, arrhythmia, and fatigue. The meta-analysis with a random model showed that the adverse events of QQC plus metoprolol treatment was lower than metoprolol treatment alone (*n* = 8 trials, RR: 0.48, 95%CI: 0.28 to 0.81, *P*=0.006, heterogeneity *χ*^2^ = 16.47, *P*=0.02, *I*^2^ = 58%, Figure 15(a)). There was no significant differences between the group treated with QQC plus trimetazidine and the group treated with trimetazidine alone (*n* = 3 trials, RR: 0.83, 95%CI: 0.26 to 2.67, *P*=0.76, heterogeneity *χ*^2^ = 0.77, *P*=0.68, *I*^2^ = 0%, Figure 15(b)).

## 4. Discussion

The characteristics of CHF are high risk of hospitalization, readmissions, mortality, morbidity, and cost. Patients with CHF suffer from a low quality of life with dyspnea, fatigue, physical exertion, mood disorders, and so on [72]. In recent years, the angiotensin-converting enzyme inhibitors, beta-blockers, aldosterone antagonists, digoxin, and diuretics as standard Western medicine have been widely used for the treatment of CHF to delay the development of myocardial remodeling [10]. However, owing to the adverse reaction, poor compliance, lower heart rate, and so on, the desired application of these drugs is restricted [2]. The combination of TCM with western medicine has been developed as a novel therapeutic approach for the treatment of CHF. It has the unique advantage of reducing the adverse reactions compared with Western medicine alone [21]. In this study, we revealed that compared with Western medicine alone, the combination of QQC with Western medicine for CHF showed better clinical efficiency. However, more evidence was required to validate the advantage of QQC plus Western medicine on reducing the occurrence of adverse effects.

QQC is a traditional Chinese medical formulation, which has been demonstrated to improve cardiac function and urine volume of CHF patients. Previous studies have found that the possible mechanisms of QQC in the treatment of CHF might be connected with reversing the increases of both AQP2 and pS256-AQP2 expression and involving the inhibition of V2R and AT1R [13, 73]. The research of the effects of QQC on cardiac function in rats with myocardial infarction suggested that it improved cardiac function by keeping the balance between proinflammatory and anti-inflammatory of cardiomyocytes [74]. The LVEF, LVEDD, LVEDV, LVESD, BNP, NT-proBNP, Hs-cTnT, and 6MWT are important indicators to reflect the cardiac function of CHF patients [17, 72, 75]. The results of this research showed that compared with Western medicine alone, the combination of QQC with Western medicine exerted positive effects on improving efficacy of CHF, with increased level of LVEF, LVEDD, and 6MWT and reduced level of HR, LVESD, BNP, and Hs-cTnT of patients with CHF. This research also suggested that QQC could improve the quality of life in patients with CHF, which was in agreement with the research in 2016 [21].

In our research, there is high heterogeneity in the meta-analysis of LVEDV, BNP, NT-proBNP, Hs-cTnT, 6MWT, and adverse effects. The level of cardiac function index is influenced by the age and gander. Moreover, etiology (type 2 diabetes mellitus, obesity, valvular heart disease, and others) and underlying disease (depression, kidney disease, sleep disordered, and others) also significantly affected the cardiovascular system [76]. These factors have not been strictly described and controlled in the included studies, which may lead to the high heterogeneity in the meta-analysis of LVEDV, BNP, NT-proBNP, Hs-cTnT, 6MWT, and adverse effects.

This research comprehensively identified the relevant literature, developed evaluation plans, and strictly implemented those plans. These methodological advantages can improve the accuracy and clinical applicability of the results of this study. However, there are several limitations in this research: (1) few studies included for meta-analysis of each indicator may lead to publication bias; (2) the description of allocation concealment is inadequate; (3) the blinding of participants personnel and outcome assessment of included studies are unclear; (4) the overall risk of bias in the included studies was generally high; (5) all subjects included in this study belong to China, it cannot account for ethnic and regional differences. Therefore, more high quality studies were required to find more convincing proof. We recommend researchers report in full their trial methodology such as random sequence generation, allocation concealment, and internationally recognized diagnosis of CHF in future publications.

## 5. Conclusion

This meta-analysis demonstrated that, compared with Western medicine alone, the combination of QQC and Western medicine exerted more positive effects on improving efficacy and increasing the level of LVEF, LVEDD, and 6MWT, as well as reducs the adverse effects and the level of HR, LVESD, BNP, Hs-cTnT of patients with CHF. The results further proved the efficacy and safety of QQC for CHF patients. However, there are several limitations in our included studies, and more high quality studies are required to provide more convincing proof.

## Figures and Tables

**Table 1:** Characteristics of included studies.

ID (Author year)	Intervention (T/C)	Sample size (T/C)		Age (T/C)	Gender (men/women)-T	Gender (men/women)-C	Outcome indicator	Course of treatment	Reference
Liu HL 2008	QQC + digoxin/digoxin	50/36		76 ± 11			Clinical efficacy, LVEF, CO, CI, SV, 6MWT, adverse reaction	4 w	[23]
Ding XZ 2011	QQC + sodium nitroprusside/sodium nitroprusside	44/40			38/6	33/7	Clinical efficacy, LVEF, LVEDD, BNP, FS		[2]
Chen WQ 2012	QQC + trimetazidine/trimetazidine	60/60		58 ± 14/57 ± 14	36/24	35/25	Clinical efficacy, LVEF, LVEDD, 6MWT, FS, E/A, adverse reaction	6 m	[24]
Huang XM 2012	QQC + sodium nitroprusside/sodium nitroprusside	39/21		59 ± 6/60 ± 5	20/10	19/11	Clinical efficacy, LVEF, LVEDD, LVESD		[25]
Zhou FZ 2013	QQC + Trimetazidine/Trimetazidine	19/20					Clinical efficacy, LVEF, CO, SV, LVESV, LVEDV	12 w	[26]
Huang XY 2013	QQC + trimetazidine/trimetazidine	30/30		65.5 ± 5.5/64.5 ± 2.5	15/15	17/13	Clinical efficacy, LVEF, LVEDD, BNP	3 m	[27]
Zhen XJ 2013	QQC + sodium nitroprusside/sodium nitroprusside	50/50		59.2 ± 5.2/59.8 ± 4.1	23/27	27/23	Clinical efficiency	8 w	[28]
Du XC 2013	QQC + sodium nitroprusside/sodium nitroprusside	56/56		63.5 ± 3.6			Clinical efficacy, LVEF, LVEDD, LVESD, MLWHF		[29]
Guo L 2014	QQCs + benazepril hydrochloridec/benazepril hydrochloridec	35/35		66/67	17/18	18/17	Clinical efficacy, LVEF, LVEDD, SBP, DBP, HR, E/A, adverse reaction	18 m	[30]
Feng CY 2015	QQC + Carvedilol/Carvedilol	30/30		58 ± 15/57 ± 18	18/12	19/11	Clinical efficacy, LVEF, LVEDD, adverse reaction	6 m	[31]
Wei HC 2015	QQC + trimetazidine/trimetazidine	42/42		64.12 ± 8.68/63.57 ± 8.54	20/22	19/23	Clinical efficacy, NT-proBNP, NO, APN	6 m	[32]
Yin YS 2015	QQC + trimetazidine/trimetazidine	42/42		64 ± 9/64 ± 8	20/22	19/23	Clinical efficacy, LVEF, LVEDD, LVESD, 6MWT, HR	6 m	[33]
Li WH 2015	QQC + trimetazidine/trimetazidine	41/41			25/16	23/18	Clinical efficacy, LVEF, LVEDD, NT-proBNP, adverse reaction	8 w	[34]
Zhang HY 2015	QQC + trimetazidine dihydrochloride/trimetazidine dihydrochloride	49/49			25/24	27/22	Clinical efficacy, LVEF, 6MWT, NT-proBNP	5 m	[35]
Zhang XW 2016	QQC + bisoprolol/bisoprolol		40/40	61.6 ± 7.4/62.7 ± 8.1	14/26	16/24	Clinical efficacy, BNP, SBP, DBP, adverse reaction	3 m	[36]
Gai YB 2016	QQC + metoprolol tartrate/metoprolol tartrate		30/30	48.12 ± 5.12/48.56 ± 4.25	15/15	18-Dec	Clinical efficacy, BNP, SBP, DBP, clinical efficacy, HR, Hs-cTnT, adverse reaction	3 m	[37]
Wang ZX 2016	QQC + sodium nitroprusside/sodium nitroprusside		47/47	58.5 ± 4.7/58.9 ± 4.6	27/20	26/21	Clinical efficiency	2 w	[38]
Sun FR 2016	QQC + sodium nitroprusside/sodium nitroprusside		34/34	64.9 ± 5.6/65.2 ± 6.8	15/19	17/17	Clinical efficacy, LVEF, plasma ADH value	2 w	[39]
Hu S 2017	QQC + metoprolol/metoprolol		62/63				Clinical efficacy, LVEF, BNP, HR	12 m	[40]
Han CX 2017	QQC + sodium nitroprusside/sodium nitroprusside		47/47	52.19 ± 5.17/53.31 ± 5.43	25/22	26/21	Clinical efficacy, LVEF, LVEDD, LVESD, SF-36	6 m	[41]
Liu YY 2018	QQC + bisoprolol/bisoprolol		49/49	67.87 ± 7.64/66.38 ± 7.94	26/23	27/22	Clinical efficacy, LVEF, LVEDD, SV, BNP	1 m	[42]
Zhou YF 2018	QQC + carvedilol/carvedilol		43/42	52.34 ± 4.52/53.71 ± 3.24	22/21	23/19	Clinical efficacy, LVEF, SV, LVESV	6 m	[43]
Ren WW 2018	QQC + carvedilol/carvedilol		39/39	58.45 ± 5.94/53.97 ± 6.18	21/18	24/15	Clinical efficacy, LVEF, LVEDD, LVESD, ADL	6 m	[44]
Li J 2018	QQC + metoprolol/metoprolol		36/36	65.41 ± 3.62/65.32 ± 3.52	17/19	18/18	Clinical efficacy, BNP, Hs-cTnT	3 m	[45]
Wang XP 2018	QQC + metoprolol tartrate/metoprolol tartrate		30/30	70.21 ± 9.11/71.04 ± 9.35	20/10	18/12	Clinical efficacy, LVEDD, BNP, E/A, adverse reaction	6 m	[46]
Wang QM 2018	QQC + metoprolol/metoprolol		29/29	63.69 ± 12.76/63.25 ± 12.36	14/15	16/13	Clinical efficacy, LVEF, HR	8 w	[18]
Liu LP 2018	QQC + trimetazidine/trimetazidine		50/50	63.3 ± 7.9/64.5 ± 7.5	26/24	25/25	Clinical efficacy, LVEF, LVESV, LVEDV	6 m	[47]
Wang XF 2018	QQC + trimetazidine/trimetazidine		46/46	61.85 ± 4.33/61.47 ± 5.14	26/20	25/21	Clinical efficacy, LVEF, LVEDD, copeptin, Gal-3, LVEDV	6 m	[48]
Yuan YZ 2018	QQC + trimetazidine/trimetazidine		47/47	60.14 ± 11.28/61.27 ± 11.41	24/23	26/21	Clinical efficacy, MLWHF, copeptin, Gal-3, adverse reaction	4 w	[49]
Wang YL 2018	QQC + ivabradine/ivabradine		51/51	55.09 ± 7.41/54.63 ± 7.92	32/19	30/21	Clinical efficacy, HR, adverse reaction	3 m	[50]
Wang CF 2018	QQC + levocarnitine/levocarnitine		40/40	70.39 ± 8.43/71.23 ± 9.95	23/17	23/17	Clinical efficacy, 6MWT, IL-6, IL-10, TNF-a	1 m	[51]
Lv Y 2018	QQC + sodium nitroprusside/sodium nitroprusside		40/40	64/66	19/21	22/18	Clinical efficacy, LVEF, LVEDD, BNP	2 m	[52]
Zhang M 2019	QQC + bisoprolol/bisoprolol		39/39	61.7 ± 2.1/62.5 ± 2.3	20/19	21/18	Clinical efficacy	3 m	[53]
Wu B 2019	QQC + coenzymeQ10+/coenzymeQ10		38/33	56.01 ± 13.55/55.12 ± 12.12	23/15	18/15	Clinical efficacy, LVEF, LVEDD, 6MWT, NT-proBNP, adverse reaction	2 m	[54]
Yang QY 2019	QQC + carvedilol/carvedilol		39/39	67.2 ± 0.6/67.4 ± 0.3	22/17	21/18	Clinical efficacy, LVEF, LVEDD, LVESD, SF-36, ADL	9 m	[55]
Meng JC 2019	QQC + carvedilol/carvedilol		53/53	66.49 ± 5.37/67.31 ± 5.24	33/20	31/22	Clinical efficacy, LVEF, CI, NT-proBNP, APN	3 m	[56]
Tan X 2019	QQC + metoprolol/metoprolol		41/41	59.4 ± 11.5/58.8 ± 12.4	20/21	22/19	Clinical efficacy, LVEF, BNP, HR, Hs-cTnT, adverse reaction	8 w	[19]
Lv DS 2019	QQC + metoprolol/metoprolol	42/42		70.56 ± 9.68/70.62 ± 9.06	25/17	28/14	Clinical efficacy, LVEF, BNP, HR, adverse reaction	6 m	[57]
Liu L 2019	QQC + metoprolol/metoprolol	40/40		62.1 ± 9.2/61.4 ± 9.3	26/14	25/15	Clinical efficacy, LVEDD, BNP, HR, E/A	6 m	[58]
Liu Y 2019	QQC + metoprolol/metoprolol	39/39		62.01 ± 5.62/61.68 ± 5.79	17/22	18/21	Clinical efficacy, LVEF, LVEDD, CI, adverse reaction	60 d	[59]
He W 2019	QQC + metoprolol tartrate/metoprolol tartrate	53/53		69.71 ± 1.10/69.51 ± 1.28	30/23	29/24	Clinical efficacy, LVEF, HR, adverse reaction	6 m	[60]
Wang L 2019	QQC + sacubitril valsartan sodium tablets/sacubitril valsartan sodium tablets	57/57					Clinical efficacy, LVEF, 6MWT, NT-proBNP	1 m	[61]
Zhou Y 2019	QQC + ivabradine/ivabradine	30/30		55.12 ± 7.99/54.98 ± 8.07	17/13	18/12	Clinical efficacy, ST2, LVEF, BNP, 6MWT, HR, Gal-3, adverse reaction	3 m	[62]
Zhang XN 2019	QQC + sodium nitroprusside/sodium nitroprusside	46/46		58.36 ± 5.24/56.75 ± 5.36	20/26	22/24	Clinical efficacy, LVEF, LVEDD, LVESD, SF-36	2 m	[63]
Li SJ 2020	QQC + coenzymeQ10/coenzymeQ10	92/92		67.0 ± 4.7/66.1 ± 4.9	48/44	51/41	Clinical efficacy, LVEF, LVEDD, SV, copeptin, Gal-3	1 m	[64]
Guo C 2020	QQC + enalapril maleate and folic acid tablets/enalapril maleate and folic acid tablets	56/56		65.2 ± 13.6/64.3 ± 11.7	32/24	31/25	Clinical efficacy, LVEF, LVEDD, LVESD, SV, BNP, 6MWT, hs-CRP, adverse reaction	8 w	[65]
Lu C 2020	QQC + metoprolol tartrate/metoprolol tartrate	53/53		62.19 ± 9.82/62.54 ± 9.11	30/23	32/21	Clinical efficacy, LVEF, 7, BNP, 6MWT, Gal-3, HO-1, hcy, adverse reaction	12 w	[20]
Su CB 2020	QQC + sacubitril valsartan sodium tablets/sacubitril valsartan sodium tablets	37/37		59.06 ± 8.52/58.39 ± 8.06	22/15	21/16	Clinical efficacy, ST2, LVEF, CO, SV, NT-proBNP	1 m	[66]
Xu JF 2020	QQC + carvedilol + sacubitril valsartan sodium tablets/carvedilol + sacubitril valsartan sodium tablets	50/50		53.79 ± 4.46/54.13 ± 7.42	22/28	27/23	Clinical efficacy, LVEF, LVEDD, LVESD, 6MWT, NT-proBNP, MLWHF	12 w	[67]
Li CY 2020	QQC + sacubitril valsartan sodium tablets/sacubitril valsartan sodium tablets	35/35		38.5 ± 4.1/35.2 ± 3.9	13/22	18/17	Clinical efficacy, LVEF, LVEDD, LVESD, BNP, Hs-cTnT, adverse reaction		[68]
Wang L 2020	QQC + levocarnitine/levocarnitine	32/32		64.21 ± 4.30/65.31 ± 5.46	18/14	17/15	Clinical efficacy, LVEF, BNP	1 w	[69]
Liu CY 2020	QQC + ;evocarnitine/levocarnitine	39/39		70.23 ± 4.65/69.78 ± 4.52	22/17	21/18	Clinical efficacy, LVEF, LVEDD	1 m	[70]

QQC: Qili Qiangxin capsule; ST2: growth stimulation expressed gene 2; LVEF: left ventricular ejection fractions; LVEDD: left ventricular end-diastolic dimension; LVESD: left ventricular end-systolic diameter; CO: carbon monoxide; CI: cardial indexes; SV: stroke volume; BNP: brain natriuretic peptide; 6MWT: 6-min walk test; NT-proBNP: N-terminal pro-B-type natriuretic peptide; NO: nitric oxide; FS: left ventricular short-axis shortening rate; SF-36: the MOS item short from health survey; ADH: antidiuretic hormone; MLWHF: Minnesota Living with Heart Failure; SBP: systolic blood pressure; DBP: diastolic blood pressure; LVESV: left ventricular end-systolic volume; ADL: activities of daily living; APN: adiponectin; hs-CRP: hypersensitive C-reactive protein; HR: heart rate; Hs-cTnT: high-sensitivity cardiac troponin T; Gal-3: galectin-3; HO-1: heme oxygenase-1; Hcy: homocysteine; LVEDV: left ventricular end-diastolic volume; IL-6: interleukin-6; IL-10: interleukin-10; TNF- *α*: tumor necrosis factor-*α*

## Data Availability

All datasets used and analyzed during this study can be available from the corresponding author upon reasonable request.

## Supplementary Materials

Supplementary Materials The PRISMA 2020 checklist for the meta-analysis.
